# Tackling Anemia in Pregnant Women in India: Reviewing the Obstacles and Charting a Path Forward

**DOI:** 10.7759/cureus.43123

**Published:** 2023-08-08

**Authors:** Rajoshee R Dutta, Pratyaksh Chhabra, Tanishq Kumar, Abhishek Joshi

**Affiliations:** 1 Medicine, Jawaharlal Nehru Medical College, Datta Meghe Institute of Higher Education and Research, Wardha, IND; 2 Community Medicine, Jawaharlal Nehru Medical College, Datta Meghe Institute of Higher Education and Research, Wardha, IND

**Keywords:** iron deficiency anemia, iron and folic acid, maternal and infant mortality, anemia mukt bharat, low birth weight babies, high-risk pregnancy, severe anemia in pregnancy, pregnant females

## Abstract

This study examined the obstacles and factors influencing the prevention and treatment of anemia among pregnant women in India. Maintaining antenatal care is essential, leading to favorable birth outcomes and healthier offspring. However, inadequate consumption of essential nutrients is widespread among pregnant women, particularly in lower and middle-income economies such as India, contributing to high maternal and infant mortality rates. The factors influencing anemia prevention and treatment are categorized into individual, socioeconomic, interpersonal, and organizational levels. This study discussed the prevalence of anemia among pregnant women in different states of India. It highlights the interventions and initiatives the government and World Health Organization (WHO) have implemented to address the issue while also emphasizing the need for comprehensive approaches that effectively address the multiple levels of influence needed to prevent and treat anemia. It calls for increased awareness, improved education, and better healthcare services to ensure proper nutrition and iron supplementation. Strengthening healthcare systems and involving family members and healthcare providers in supporting pregnant women are crucial for successful anemia prevention and treatment programs.

## Introduction and background

The advantages of maintaining proper nutrition, especially during antenatal care, are well established. Pregnant mothers consuming a healthy and balanced diet leads to favorable birth outcomes and healthier offspring. In lower and middle-class income nations, inadequate consumption of nutrients, such as vitamins, minerals, proteins, and iron and folic acid (IFA), according to the acceptable values for both mother and the baby, is widespread [1]. According to the National Family Health Survey-4 (NFHS-4), one out of every four women in India has an undernourished diet, with a body mass index (BMI) lower than 18.5 kg/m^2^ causing deficiencies such as iron deficiency anemia. Providing nutrition supplements to mothers in the form of IFA tablets is the most efficient and scalable intervention, which reduces the burden of undernutrition and has been shown to impact various health outcomes positively [2].

In accordance with the World Health Organisation's classification of anemia, pregnant females whose hemoglobin (Hb) levels are equal to or greater than 11 g/dL are categorized as non-anemic. Those with Hb levels ranging from 10.0 to 10.9 g/dL are classified as mild anemic, while individuals with Hb levels between 7.0 and 9.9 g/dL are deemed moderate anemic. Lastly, those with Hb levels below 7.0 g/dL are classified as severe anemic. India has implemented several health programs over the years to prevent anemia, but despite these efforts, over 50% of pregnant women are still affected by the same. This study examines the obstacles preventing anemia and assesses the attitudes and behaviors of women with anemia towards their condition [3].

## Review

Methodology

Eligibility Criteria

The eligibility criteria included all review articles and original studies which discussed the factors influencing anemia in pregnant women. Review articles that discussed basic concepts of anemia and strategies employed by the government to tackle pregnancy were also included. Articles and studies which discussed anemia in children or men were excluded. There were no other exclusion criteria. 

Literature Search Strategy

The literature search was conducted by all the authors. The PubMed electronic database was used for the literature search. The duration of publications searched included articles from the year 2011 to the year 2023, and the last search was done on July 2, 2023. A literature search was conducted using the key terms: "anemia," "pregnancy," "maternal mortality," "infant mortality," "Anemia Mukt Bharat," "low-weight babies,", "high-risk pregnancy," and "iron deficiency anemia." These were combined with adjuncts of "AND" as well as "OR" to review specific subtopics of the article.

Data Extraction

**Figure 1:**
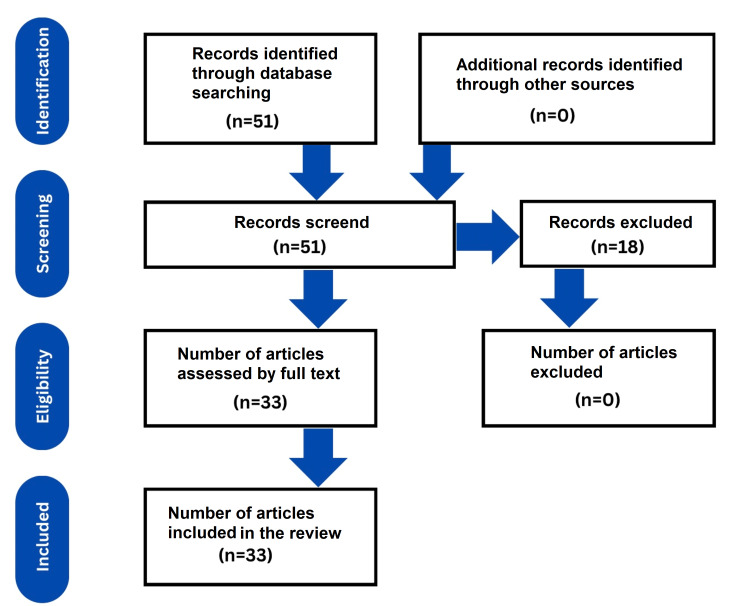
PRISMA flow diagram for screening and selecting articles for factors influencing anemia in pregnant women. PRISMA: Preferred Reporting Items for Systematic Reviews and Meta-Analyses

The abstracts of the articles generated from the literature search were reviewed by all authors independently. Those who met the selection criteria were studied and assessed for their full texts. The search process is demonstrated in the flow chart in Figure 1.

Interventions taken by the government

The government of India and the Word Health Organization (WHO) have been collaborating on India's primary health concerns, which include anemia. According to statistics from the National Family Health Survey (NFHS-5), 52.2% of pregnant women in the country between the ages of 15 and 49 years are considered anemic. The states with the highest prevalence are Ladakh (78.1%), Bihar (63.1%), Gujarat (62.6%), West Bengal (62.3%), and Odisha (61.8%). The primary duty of enhancing healthcare services which encompass the execution of National Health Programs rests with the state/union territories (UT) government in question. Nevertheless, the Ministry of Health and Family Welfare offers financial and technical support through the National Health Mission (NHM).

The Anemia Mukt Bharat (AMB) initiative, which the Indian government introduced in 2018, aims to eliminate anemia in mothers, children, and adolescents using a life cycle approach. An integrated approach has been implemented to combat anemia in specific populations, focusing mainly on adolescent girls and pregnant women. The strategies include targeted interventions such as providing preventive IFA supplements to girls aged between 10 and 19 years, conducting behavior change communication campaigns, promoting delayed cord clamping, and utilizing digital hemoglobinometers as a point of care testing (POCT) for immediate anemia testing and treatment.

Addressing factors beyond nutrition, such as malaria, hemoglobin disorders, and fluorosis, is also emphasized in specific areas. Severe anemia in pregnant women is managed through intravenous administration of iron sucrose or blood transfusion, while healthcare workers in high-priority districts are incentivized to identify and monitor cases. Additionally, a national deworming program is conducted twice a year, including children aged 1-19 years, women of reproductive age, and pregnant women. Medical officers and frontline workers receive training on updated guidelines to ensure effective implementation, while Accredited Social Health Activists (ASHAs) engage in community-level awareness campaigns. These anemia case management interventions for expectant mothers under AMB include Rajasthan, Madhya Pradesh, Haryana, Jharkhand, and Bihar [4].

Factors 

**Figure 2:**
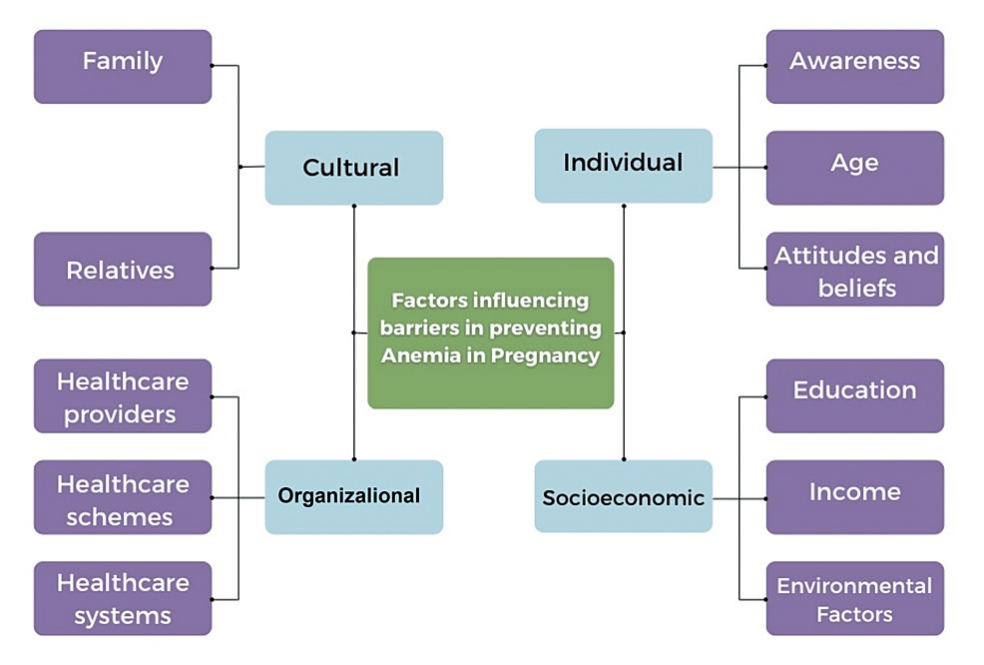
Factors influencing barriers in preventing anemia in pregnancy.

In order to understand the barriers to preventing anemia, we have compiled the variables into a model. The model is divided into four broad categories as follows: (1) individual, (2) socioeconomic, (3) cultural, and (4) organizational. Figure 2 represents the variables that influence barriers to preventing pregnancy. It has been divided into the four categories mentioned above and subdivided into individual factors.

These theories acknowledge that a person's interpersonal interactions, immediate surroundings, and systems impact their behavior and health. The theory discusses many levels of influence, including individual, cultural, and organizational aspects, that may be acted upon to influence the quality of life. Each of these impact levels significantly influences health [5].

Individual factors

Awareness

According to a study conducted in India published in 2021 consisting of 210 anemic mothers, 35.2% had knowledge about iron requirements during pregnancy, 74.8% had knowledge about iron supplement tablets, and 35.2% had knowledge about iron tablets provided free of cost by the government. Participants agreed that "eating well" was crucial. They also agreed that leafy vegetables, beetroot, jaggery, pomegranate, spinach, and carrots are rich in iron. The expense of food proved a barrier to good eating, in any case [5,6]. Another study conducted in Puducherry and published in 2020 discovered that participants had a "moderate" understanding of the requirement for iron to improve blood count and knowledge of anemia prophylaxis. Several mothers reported taking iron supplements due to the health professionals' emphasis on delivering health information [7].

Age

According to a study conducted in India, it was observed that anemia was common in pregnant women in the age group of 21-30 years [8]. Risk factors for such women consist of poor education, poor economic circumstances, and lower body mass index, which significantly increases the likelihood of anemia [9]. Additionally, the greater likelihood of anemia among adolescent women may be attributed to unforeseen pregnancy and poor physical conditions before conception, resulting in a higher prevalence of anemia than older women [10].

Attitudes

According to research done in India, these women had different views on anemia; 54.7% believed that gaining weight is vital for a healthy pregnancy, and 48.1% believed pallor indicated anemia needs treatment. Only 20.9% believed being easily fatigued and having shortness of breath was worrisome. A total of 61.9% of women boosted their consumption of calcium, folic acid, and iron. Only 9.5% of pregnant women had their hemoglobin measured throughout the first trimester and continued taking regular iron supplements.

Ninety percent of the participants in this trial did not take their iron supplements as directed. A total of 21.5% were unable to comply owing to insufficient supplies, while 20% were unable to comply due to forgetfulness. Due to adverse effects, including constipation (4.7%) and gastritis (5.7%), many women refused to maintain consistency [6].

Socioeconomic factors

Education

In recent literature, women's education level has been documented to account for the general understanding of reproductive health programs and accommodative policies, including supplemental nutrition [11]. Tanzanian research found that women with secondary or higher education had a lower anemia burden (18.0%) than those with no formal education (23.4%) or only primary education (58.6%) [12].

Pregnant women with greater education levels tend to have anemia less frequently. According to our findings, a 1% increase in higher education is associated with a 1.12% decrease in the prevalence of anemia. Anemia decreases by 0.001% for every 1% rise in educational level [13].

Income

Lower income is believed to predispose to anemia in pregnancy [14]. According to research done in Punjab in 2020, out of 500 women, 350 women with anemia had incomes under rupees 10,000, compared to 34 who had incomes between 10,000 and 20,000, and 25 who had incomes beyond 20,000. The prevalence of anemia was higher in participants (85.6%) with monthly incomes under 10,000 per capita. People in low socioeconomic classes are more likely to be uneducated and frequently face financial difficulties. It is further confirmed by the finding that low-income women frequently eat meals deficient in minerals, proteins, and vitamins [15].

Environmental Factors

**Figure 3:**
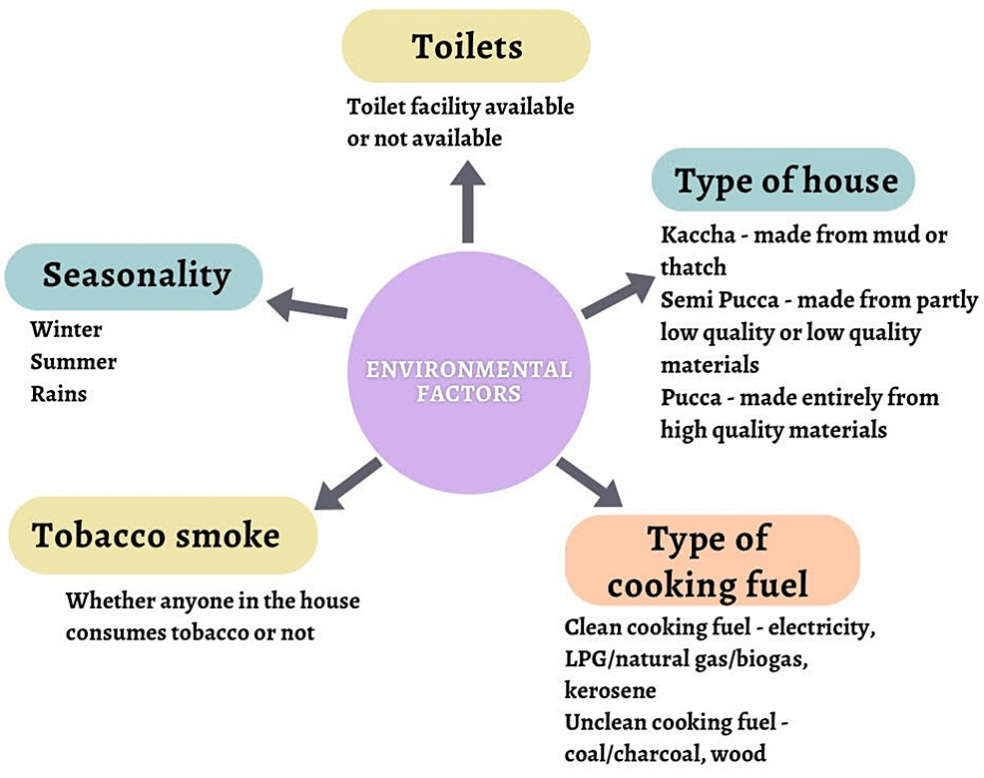
Various environmental factors linked to anemia based on the following five main components: toilets, type of house, type of cooking fuel, tobacco smoke, and seasonality.

Environmental variables consist of five main components as follows: toilets, the type of house, the type of cooking fuel, tobacco smoke, and seasonality. According to a study conducted in 2014, which explores the association between household environment and the prevalence of anemia in India, the prevalence of anemia was higher among households with no toilet facilities. The type of house also made a meaningful impact, where higher prevalence was seen among those in kaccha or semi-pucca houses. People who lived in homes with unclean fuels had a higher prevalence of anemia than those who lived in homes with clean fuel. A higher number of people who reported having anemia symptoms had exposure to tobacco smoke at home, compared to people who had no exposure to tobacco smoke at all. The seasons also influenced anemia; it was more common in the summer and during the rainy season than in the winter [16]. Figure 3 below shows various environmental factors linked to anemia based on the five main components mentioned above.

Cultural factors

Nuclear households which are usually defined as family units that consist of a married couple along with their children, were shown to have a much greater prevalence of anemia. This may be due to a lack of family members in nuclear families who could care for expectant mothers or due to their busy schedules. As a result, expectant mothers might not receive adequate care for diet and nutrition [17].

In unusual cases, the mother's inability to take iron supplements may be caused by familial beliefs. According to research, the mothers said their relatives had cautioned them against taking IFA supplements, typically because they feared that doing so would make the fetus grow too big and complicate delivery [5].

In a behavioral study about perceptions of antenatal iron-folic acid supplements conducted in 2014, it was found that in nuclear families, husbands assist their wives by contributing around the house and encouraging them to eat healthily [18].

Future studies must determine the best ways to involve spouses, mothers-in-law, and sisters-in-law as supporters of anemia prophylaxis and compliance to therapy, such as appointments with the ASHA worker or a physician. Giving husbands designated tasks or responsibilities for helping their spouses would help significantly [19].

Organizational factors

Healthcare Providers

In India, pregnant women typically receive IFA supplements from ASHAs, Anganwadi workers (AWW), and auxiliary nurse midwives (ANM). Among these workers, auxiliary nurse midwives are stationed at a primary health care center (PHC). Anganwadi workers, on the other hand, operate solely within their villages and primarily focus on providing nutritious food as well as supplements to children, young girls, and women (especially those who are pregnant and lactating). Similarly, ASHA workers serve their appointed village, especially pregnant women and children. They keep track of immunization, supplementation as well as facilitate deliveries. It is also important to note that if women are diagnosed with anemia, they can obtain free IFA supplements from government establishments, regardless of their pregnancy status [20].

Based on studies, it was found that if the following information was provided to women by their healthcare providers, it increased awareness and compliance toward anemia treatment: (1) their hemoglobin (Hb) level during pregnancy and breastfeeding, (2) the adverse effects of anemia during pregnancy and on the developing fetus, and (3) the side effects of iron and folic acid supplements [21,22].

Healthcare Schemes

To address the current state of anemia among all specified populations, the Indian government has launched several initiatives. The government offers financial and technical support for the Anemia Mukt Bharat strategy's execution through the National Health Mission. The government has developed several programs to ensure this. Table 1 given below highlights these programs.

**Table 1:** The following table lists out the major interventions taken by the government under Anemia Mukt Bharat strategy. SUMAN: Surakshit Matritva Aashwasan; JSY: Janani Suraksha Yojana; JSSK: Janani Shishu Suraksha Karyakram; LaQshya: Labour Room Quality Improvement Initiative; RMNCH+A: Reproductive Maternal Newborn Child Health and Adolescent Health Services; MCP Card: Mother Child Protection Card; RCH: Reproductive and Child Health; VHSND: Village Health Sanitation Nutrition Day; ICDI: Integrated Child Development Services

Initiatives	Description	Source
Surakshit Matritva Aashwasan (SUMAN)	SUMAN aims to provide guaranteed, dignified, respectful, and high-quality healthcare at no cost to every mother and newborn visiting public facilities to eliminate avoidable maternal and newborn fatalities	[23]
Janani Suraksha Yojana (JSY)	JSY is a program that promotes institutional delivery through conditional cash transfers	[24]
Janani Shishu Suraksha Karyakram (JSSK)	JSSK ensures that every pregnant woman is entitled to free childbirth at public health facilities, including cesarean sections, along with accessible transportation, diagnostic tests, medications, and diet	[25]
Labour Room Quality Improvement Initiative (LaQshya)	LaQshya improves the standard of care in labor rooms to ensure kind and respectful care during delivery and the initial weeks after childbirth	[26]
Anganwadi centers	Anganwadi centers participate in a monthly Village Health Sanitation Nutrition Day (VHSND) aligned with the Integrated Child Development Services (ICDI)	[25]
Delivery locations	Over 24,000 delivery points nationwide are established to provide comprehensive Reproductive, Maternal, Newborn, Child Health, and Adolescent Health Services (RMNCH+A)	[4]
MCP cards and safe motherhood pamphlets	Expectant mothers receive MCP cards and safe motherhood pamphlets to educate them about deliveries in healthcare facilities, signs and symptoms of pregnancy, proper diet, and rest	[4]
Reproductive and Child Health (RCH)	The RCH portal offers internet-based tracking for expectant mothers and comprehensive prenatal and postnatal care	[4]

It was found that, in general, participants received free IFA supplements from hospitals. Some women from Karnataka bought supplements from stores. In Uttar Pradesh, ASHA workers provided supplements, and National Iron Plus Initiative (NIPI) supported anemia reduction. Karnataka also had related initiatives such as Matru Poorna Yojana but not any specific anemia program [26].

Healthcare Systems

The World Health Organization (WHO) aims to achieve a worldwide target of reducing anemia in women in the reproductive age group by 50% by 2025. In order to achieve this goal, it is crucial to prioritize addressing anemia during pregnancy and breastfeeding [27].

A study conducted in Dwarka in 2022 revealed that using nutritional and health services in primary health centers could have been better. Although most women surveyed received either antenatal care (ANC) or postnatal care (PNC), there were limitations regarding comprehensive ANC and PNC. PNC or ANC services were not utilized by approximately 15% of women. Similar results were found in Delhi [28] and Uttar Pradesh [29,30].

ANC and PNC are essential services that women during pregnancy and postpartum should receive as part of comprehensive health programs. According to WHO, it is recommended to have at least four antenatal visits which should occur around 16 weeks, 24th-28th weeks, 32 weeks, and 36 weeks. Additionally, there should be a minimum of four postnatal visits within 6-12 hours after delivery. Visits between three and six days, six weeks, and six months after birth should also be conducted [31].

According to the guidelines of Integrated Management of Neonatal and Childhood Illness (IMNCI), for low birth weight (LBW) babies, it is recommended to include three to four additional postnatal care (PNC) visits on the 14th, 21st, and 28th day. Home visits effectively improve maternal and child health outcomes [32].

Home visits throughout pregnancy and after delivery can enhance the necessity for and use of antenatal and postnatal care amenities, potentially reducing Maternal Mortality Rate (MMR) and Infant Mortality Rate (IMR) by a minimum of 14-19% according to comprehensive studies carried out in African and South Asian nations [33].

## Conclusions

The prevalence of anemia among pregnant women in India remains a significant public health concern. The factors contributing to this problem are multifaceted, encompassing individual, socioeconomic, cultural, and organizational aspects. The amount of iron and folic acid consumed depends on personal characteristics like anemia awareness and attitudes. For the sake of enhancing mother and child health outcomes, education, and knowledge are essential. The risk of anemia is also influenced by age, with younger women being more susceptible than older women. Income and education are two socioeconomic factors that significantly impact anemia prevalence. Women from low educational backgrounds and households with poor incomes are more at risk. Addressing these discrepancies through targeted interventions and enhancing access to healthcare services is imperative.

Better health outcomes may be caused by cultural variables, particularly the assistance and participation of family members, particularly husbands, as supportive allies in the care of pregnant women. Organizational issues like the participation of healthcare providers and healthcare programs are essential. Healthcare systems must be strengthened, medical staff must be adequate, and IFA supplements must be readily available. In conclusion, boosting awareness, enhancing education, addressing socioeconomic disparities, fostering family support, and building healthcare institutions are all essential components of a comprehensive and multifaceted strategy to combat anemia among pregnant women in India.
